# Obesity Management: What Should We Do If Fat Gain Is Necessary to Maintain Body Homeostasis in a Modern World?

**DOI:** 10.3389/fendo.2018.00285

**Published:** 2018-06-04

**Authors:** Angelo Tremblay

**Affiliations:** Department of Kinesiology, Université Laval, Québec, QC, Canada

**Keywords:** environment, adipose tissue, body weight, pollution, sleep

## Abstract

The prevalence of overweight has substantially increased over the last decades despite the intent of health professionals and the general population to prevent this trend. Traditionally, this phenomenon has been attributed to unhealthy dietary macronutrient composition and/or to the decrease in physical activity participation. Beyond the influence of these factors, it is more than likely that other factors have influenced energy balance in a context of modernity. These include inadequate sleep, demanding cognitive effort, chemical pollution, and probably others which also have the potential to promote a positive energy balance but which are also part of the reality of success and productivity in a globalized world. As discussed in this paper, many individuals may become conflicted with themselves if they wish to prevent weight gain while influencing factors which are determinants of their socioeconomic success. In this regard, this paper reminds us of the contribution of adipose tissue gain in body homeostasis which is essential to permit energy balance, especially under lifestyle conditions promoting overfeeding. From a clinical standpoint, this imposes the consideration of a weight loss program as a search for compromise between what can be changed to promote a negative energy balance and what can be tolerated by the body in terms of fat loss. Furthermore, if we also consider the impact of pollution on energy balance for which we currently do not hold solutions of reversibility, we probably must accept that the mankind of today will have to be more corpulent than its ancestors. In this pessimistic environment, there are still possibilities to do better; however, this will probably require the revisiting of lifestyle practices according to what the human body and planet can tolerate as deviation from optimal functioning.

## Introduction

Obesity has become a major preoccupation over the last decades because of its important increase in its prevalence all around the world. This reality has obviously mobilized researchers and health professionals who have significantly improved their knowledge and skills about obesity management. However, population statistics clearly show that this progress has not been sufficient since the prevalence of obesity continues to increase in most countries, including those where famine and malnutrition have traditionally been the main food-related problems. Furthermore, currently available estimates suggest that obesity prevalence may continue to increase in the foreseeable future (1). Thus, it is not unreasonable to emphasize in the title of the special issue of this journal that obesity becomes the new normal human condition. In fact, this title is particularly relevant for the physiologist who perceives adipose tissue as an important organ for the maintenance of body homeostasis. As described in the present paper, adipose tissue contributes to the control of appetite and thermogenesis, plays an active role in the secretion of hormones involved in metabolic regulation, and acts as a storage site of lipid soluble pollutants. Thus, a physiological perception of obesity considers that excess body fat is the problem of individuals who rely too much on body fat gain to maintain adequate body functioning. According to the theme of this journal issue, the question that emerges is “What should we do when body fat gain is needed to maintain body homeostasis in today’s life?” To document this issue, four proposals of responses are presented about “what we should do” together with some relevant implications.

## Proposal 1: To Better Understand the Determinants of Obesity and to Intervene According to Relevant Causes

It is a truism to indicate that body fat gain happens in accordance to the first law of thermodynamics (i.e., it results from an excess energy intake over energy expenditure). This observation has prompted the simplistic deduction that body fat gain results from gluttony and laziness or, in a broader sense, from suboptimal macronutrient intakes and insufficient exercise. From a clinical standpoint, this has dictated the emergence of diet prescriptions such as low and very low calorie diets which completely disregard the tolerability of the body regarding these dietary practices. As further discussed, this ignorance of body homeostatic factors has generally resulted in weight regain to a value frequently exceeding pre-intervention body weight. In addition, recent research has highlighted a significant weight gaining effect promoted by non-traditionally considered lifestyle factors. These factors are not necessarily inducing a direct change in caloric intake or expenditure, but rather represent a source of stimuli that more discretely affect energy balance *via* an impact on some regulatory systems.

According to results of the Quebec Family Study (2), short sleep duration appears on top of the list of non-traditionally considered determinants of excess body weight. While population studies have clearly shown a secular decrease in sleep duration in many countries (3), laboratory-based studies have documented a clear link between insufficient sleep and the proneness to a positive energy balance, as described in Table 1. In addition, our research experience has showed that overweight is particularly pronounced in the short sleeper when it is accompanied by a high disinhibition eating behavior profile being related to reduced appetite control and food overconsumption (4).

**Table 1:** Summary of the effects of short sleep duration on energy balance and related variables.

Effect	Reference
Reduces the plasma concentration of the anorectic hormone leptin	Spiegel et al. (5); Chaput et al. (6)
Increases the plasma concentration of the orexigenic hormones ghrelin and cortisol	Spiegel et al. (7); Spiegel et al. (5)
Promotes mild hypoglycemia	Chaput et al. (8)
Increases spontaneous energy intake	Brondel et al. (9)
Reduces spontaneous physical activity	Schmid et al. (10)
Interferes with the outcome of a diet restriction on body fat	Nedeltcheva et al. (11); Chaput and Tremblay (12)

Beyond the impact of inadequate sleep habits on energy balance and body composition, many other non-traditional determinants of overweight seem to be influential in a modern lifestyle. These include persistent organic pollutants (13–15), inadequate feeding behaviors (16–18), demanding cognitive effort (19, 20), and suboptimal micronutrient intake (21), especially low calcium intake (22–24). As emphasized above, these factors are not vectors of high caloric input or output but like for sleeping, they exert a significant impact on regulatory mechanisms affecting energy balance.

From a clinical standpoint, “what should we do?” must focus on actions directly targeting factors promoting excess energy intake. For instance, if short sleep duration or low sleep quality appears to have a causal link with the body weight status of a patient, the primary focus of clinical actions should target optimal sleep habits. On the other hand, it might well be counter-productive to rely on traditional approaches such as diet restriction and physical activity to intervene in this case. After all, exercise does not seem the optimal remedy for people who are vulnerable because of fatigue and the lack of adequate body recovery.

The multifactorial nature of the environmental determinism of excess energy intake also emphasizes the necessity to broaden multidisciplinary collaboration in the management of obesity. This *a priori* requires a more detailed evaluation and characterization of obese individuals before implementing an obesity management program. If successful, this approach should permit a more individualized management, possibly *via* the use of new technologies, that should contribute to the well-being of individuals as well as their success in body weight loss and maintenance.

## Proposal 2: To be Preoccupied by an Adequate Balance Between Physical and Cognitive Effort

A discussion pertaining to obesity rarely challenges the adequacy of the socioeconomic context to which most people are confronted with today. As previously described (25, 26), we now have to deal with a globalized environment for which key words like performance and productivity are the main targets for many workers. This reality has also been accompanied by changes in the modalities of daily labor which now mostly relies on cognitive effort instead of physical work. From a biological standpoint, there is a major difference in the metabolic flexibility that is allowed to physical and mental work. Physical work relies on muscle cells that have the flexibility to use carbohydrate or lipid as fuel for ATP production. Furthermore, they also have the possibility to accentuate the use of a substrate versus the other one depending on carbohydrate availability. This is in contrast with mental work that is based on the contribution of neurons which depend on carbohydrate availability for their activity. In this case, metabolic flexibility is reduced and the resulting work may depend more acutely on glucose availability. This observation guided us toward the hypothesis according to which prolonged demanding cognitive effort would increase energy intake to sustain carbohydrate availability. This hypothesis was initially investigated by our team in female students at Laval University who were tested under conditions of demanding mental work which were compared to a control relaxed condition. The main results obtained in these studies are presented in Table 2. Globally, the observed effects support the idea that demanding cognitive effort has the potential to induce a substantial acute positive energy balance. This is in agreement with the results reported by McCann et al. (27) who found that episodes of high cognitive effort in researchers preparing grant applications promoted stress and hyperphagia. This is also concordant with a recent study demonstrating that long periods of stressful homework are associated with an increase in body fat in male children (20).

**Table 2:** Acute effects of demanding cognitive effort on components of energy balance and related variables.

Effect	Reference
Increase in energy intake without changes in appetite sensations	Chaput and Tremblay (19)
Very weak enhancing effect on energy expenditure	Chaput and Tremblay (19); Chaput et al. (28)
Increase in energy intake associated with increased cortisolemia and glycemia instability	Chaput et al. (29)
Decrease in parasympathetic nervous system activity	Pérusse-Lachance et al. (30)

Our research experience reveals that physical activity could represent an appropriate solution to prevent the hyperphagia induced by demanding cognitive effort. We have reported that a timely inclusion of a brief period of exercise between cognitive effort and mealtime prevents the hyperphagia induced by mental work (31). Concordant results have been recently reported by other investigators (32). This is also consistent with the success obtained with sport/study programs that contribute to improve fitness and prevent weight gain without altering academic success even if school time is reduced (33).

Another option to prevent the positive energy balance resulting from demanding cognitive effort is provided by pharmacology. This is the case for methylphenidate which is the active agent of drugs like Ritalin and Concerta. It has been shown that Ritalin reduces by about 50% the increase in carbohydrate utilization induced by mental work (34). This is in agreement with the observation that methylphenidate decreases spontaneous *ad libitum* energy intake (35). In individuals with obesity known to be resistant to weight loss, the supplementation of Concerta was found to result in substantial weight loss when compared to individuals receiving the same nutritional supervision with a placebo (36). Taken together, these observations suggest that methylphenidate helps to deal with demanding cognitive effort while preventing its hyperphagic effect. Spontaneously, this fits with the preoccupation of the health professionals treating obesity although it remains unclear if such a practice should be part of “what we should do in obesity management.”

## Proposal 3: To Recognize the Importance of the Regulatory Role of Adipose Tissue on Energy Balance and to Plan Realistic Interventions

Lipid storage is the main function that has been traditionally attributed to adipose tissue. Even if we have been aware of the existence of ectopic fat deposition, adipocytes have been the target of obesity management because of their large capacity to store lipids and to release fatty acids in the context of dietary restriction. Several decades ago, the perception of adipose tissue had to be revisited with the discovery of leptin (37) which was the first hormonal messenger secreted by fat cells that was documented for its properties to affect energy balance. Immediately after its discovery, leptinemia was found to fluctuate according to energy balance (38). Accordingly, an increase in body fat was reported to be associated with an increase in leptinemia that suggested a state of leptin resistance in obese individuals (39).

The changes of plasma leptin induced by body weight/fat loss are of particular interest for the discussion of the present paper. In this regard, our experience and that of others showed that weight loss is associated with an increase in hunger (40, 41) as well as a greater than predicted decrease in energy expenditure both in the resting (42) and active (43, 44) states. Furthermore, the observation that leptin administration to weight-reduced individuals with obesity reverses these effects (41) should be viewed as a clear proof of concept pertaining to the regulatory role of fat cell secretion on energy balance. From a clinical standpoint, these observations also suggest that messengers of adipose tissue like leptin may contribute to body weight restabilization in the context of overfeeding and to the occurrence of resistance to further lose body fat in response to a prescribed negative energy balance. This also emphasizes the relevance to use some molecules such as leptin in drug formulation to attenuate the physiological vulnerability of weight-reduced obese individuals.

What does it mean in terms of “what we should do” today in the management of obesity? From a clinical standpoint, the first implication of a concept focusing on the protective role of adipose tissue is the necessity to improve lifestyle practices to prevent fat regain in the weight-reduced obese individuals. As explained above, variations in fat mass affect both energy intake and expenditure. Thus, the weight-reduced obese individual should improve his/her food, physical activity, and sleep habits with the hope that the resulting gains in functionality will be sufficient to compensate for the impact of fat loss on energy balance. According to the experience of some members of the US National Weight Loss Registry, lifestyle changes can permit the maintenance of large body weight loss over years provided that they adhere to a new lifestyle in a weight-reduced obese state. For instance, McGuire et al. (45) found that members of the registry reporting a low-fat diet (25% dietary energy as fat) and a regular physical activity participation were able to maintain a 30 kg weight loss for 5.7 years. However, this does not mean that these new life habits will be sufficient to bring back body weight to pre-obese values. Our experience reveals that healthy eating and vigorous physical activity participation can induce a substantial weight loss up to resistance to further lose fat which was, however, not sufficient to reach baseline pre-obese body weight values (46, 47). As further discussed in the next section, we cannot exclude that humans in a modern world should accept to be more corpulent than their ancestors.

## Proposal 4: To Consider That Some Environmental Pollutants Can Interfere with a Healthy Regulation of Energy Balance and to Try to Counteract this Effect

The comfort and productivity that have been provided by modernity have at least partly been achieved *via* chemistry innovation that allowed the development of high performance chemicals. In agriculture, these compounds have been used as insecticides to improve the outcome of crops. In the industrial sector, they have been successfully used in the formulation of paints or for their insulating properties in the development of electrical transformers. In fact, their great usefulness at low price has promoted their use throughout the planet. However, we now realize that the enthusiasm that favored the dissemination of these compounds exaggeratedly dominated the knowledge of their side effects that had the potential to prevent their use. Several decades after the beginning of their commercialization, the evidence demonstrating their link with the development of hormone-dependent cancers was sufficiently strong to justify their banishment in many countries. Despite their withdrawal, their long half-life and the liposolubility of some of them confer a clear interest in the study of obesity and its complications.

According to Lee et al. (48), the old pollutants of the family of organochlorines are particularly detrimental regarding the proneness to metabolic dysfunctionality. In addition, they are of great interest for the obesity management since body fat represents the dilution space of these compounds in the body. This issue becomes a problematic matter in weight-reducing programs since body fat loss results in the increase of their concentrations in blood and tissues (13–15, 49).

About 20 years ago, we initiated a research program oriented toward the study of the fat loss-induced increase in circulating organochlorine pollutants on the energy metabolism in individuals with obesity subjected to weight loss. In brief, the strategy used consisted of measuring organochlorine concentrations in blood and adipose tissue before and after a weight-reducing program. The observed changes in concentrations were correlated with those of different biomarkers. This was the best available approach to examine the possibility of a causal link between pollutant changes and those in energy metabolism in humans. Thus, following a weight loss of about 10 kg in individuals with obesity, we observed the following adaptations that were correlated with the increase in the blood concentration of some pollutants: (1) a decrease in resting metabolic rate being significantly greater than the changes predicted by weight loss (50); (2) a decrease in weight-adjusted sleeping metabolic rate being explained more by changes in blood pollutants than by those in leptinemia (51); (3) a decrease in circulating levels of thyroid hormones (50); and (4) a decrease in skeletal muscle oxidative enzymes (52). Taken together, these observations suggest that the body thermogenic capacity had become more vulnerable with weight loss possibly because of the detrimental associations with changes in blood pollutants.

The issue of “what we should do” to promote sustainable solutions is particularly challenging for the pollutant-obesity connection. It is irrelevant to hope that the natural clearance of these compounds will re-establish a rapid return of homeostasis since the half-life of some of them lasts several decades. On the other hand, since it is not realistic to omit losing weight to prevent pollutant body hyperconcentration, the stimulation of body clearance remains the only practical option of treatment. Up to now, the lipid analog Olestra has been the main documented compound for its potential detoxifying properties regarding lipid soluble pollutants. In severely contaminated individuals with TCDD (2,3 7,8-tetrachlorodibenzo-p-dioxin), this non-digestible dietary fat substitute accelerated the intestinal excretion of TCDD by 8–10 times the expected clearance which was sufficient to decrease from 7 to 1.2 years the elimination half-life of this compound (53). This is in agreement with the results reported by Redgrave et al. (54) who also demonstrated a strong detoxifying effect of Olestra in an individual subjected to a large amount of weight loss. In individuals with obesity, our experience with the use of Olestra was more limited. Indeed, following a body fat loss of about 3 kg, Olestra accentuated the clearance of β-hexachlorocyclohexane which significantly differed from its increase in blood concentrations following a comparable fat loss without Olestra. However, we did not find significant differences for changes in 18 other pollutants in response to Olestra supplementation (55).

There is also no clear evidence showing that specific dietary modifications can exert a substantial body detoxifying effect. For instance, when comparing blood organochlorine concentrations between omnivores and vegans, only small differences favoring vegans were observed between the two groups (55). Interestingly, a relationship between the concentrations of body pollutants and some gut bacteria was also recently reported (56). However, this does not permit yet realistic inferences that a stimulation of the gut microbiota could favor body detoxification. Globally, these observations suggest that as far as the pollutant-obesity issue is considered, there is no clear solution that would permit some actions improving body homeostasis in a palpable manner.

## Conclusion

The evidence summarized in this paper suggests that it is maybe realistic to consider that overweight has become the new normal scenario of mankind. Indeed, the changes in our lifestyle are so deep in terms of the way of living and were so dictated by the search of comfort and well-being that there is no perspective for a spontaneous return toward a less obesogenic traditional lifestyle. On the other hand, as explained in this paper, body fat gain appears as a homeostatic adaptation that compensates for some effects of this lifestyle on some components of the body’s biology such as the regulation of energy balance. In this context where obesity seems to be a condition which clearly places many individuals in conflict with themselves, the most realistic “what we should do” is to promote the healthiest lifestyle as possible with the hope that adipose tissue compensations will be minimally solicited to permit body homeostasis.

## Author Contributions

AT is the unique author of this manuscript. Therefore, he took in charge all the sections of this paper.

## Conflict of Interest Statement

The author declares that the research was conducted in the absence of any commercial or financial relationships that could be construed as a potential conflict of interest.
